# Plasma cell subtypes analyzed using artificial intelligence algorithm for predicting biochemical recurrence, immune escape potential, and immunotherapy response of prostate cancer

**DOI:** 10.3389/fimmu.2022.946209

**Published:** 2022-12-08

**Authors:** Xiao Xie, Chun-Xia Dou, Ming-Rui Luo, Ke Zhang, Yang Liu, Jia-Wei Zhou, Zhi-Peng Huang, Kang-Yi Xue, Hao-Yu Liang, Ao-Rong Ouyang, Sheng-Xiao Ma, Jian-Kun Yang, Qi-Zhao Zhou, Wen-Bing Guo, Cun-Dong Liu, Shan-Chao Zhao, Ming-Kun Chen

**Affiliations:** ^1^ Department of Urology, The Third Affiliated Hospital of Southern Medical University, Guangzhou, China; ^2^ The Third Clinical college, Southern Medical University, Guangzhou, China; ^3^ College of nursing, Jinan University, Guangzhou, China; ^4^ State Key Laboratory of Oncology in South China, Sun Yat-sen University Cancer Center, Guangzhou, China; ^5^ Department of Urology, Nanfang Hospital, Southern Medical University, Guangzhou, China; ^6^ Department of Obstetrics and Gynecology, The Third Affiliated Hospital of Southern Medical University, Guangzhou, China

**Keywords:** prostate cancer, plasma cell, artificial intelligence, immune escape, immunotherapy

## Abstract

**Background:**

Plasma cells as an important component of immune microenvironment plays a crucial role in immune escape and are closely related to immune therapy response. However, its role for prostate cancer is rarely understood. In this study, we intend to investigate the value of a new plasma cell molecular subtype for predicting the biochemical recurrence, immune escape and immunotherapy response in prostate cancer.

**Methods:**

Gene expression and clinicopathological data were collected from 481 prostate cancer patients in the Cancer Genome Atlas. Then, the immune characteristics of the patients were analyzed based on plasma cell infiltration fractions. The unsupervised clustering based machine learning algorithm was used to identify the molecular subtypes of the plasma cell. And the characteristic genes of plasma cell subtypes were screened out by three types of machine learning models to establish an artificial neural network for predicting plasma cell subtypes. Finally, the prediction artificial neural network of plasma cell infiltration subtypes was validated in an independent cohort of 449 prostate cancer patients from the Gene Expression Omnibus.

**Results:**

The plasma cell fraction in prostate cancer was significantly decreased in tumors with high T stage, high Gleason score and lymph node metastasis. In addition, low plasma cell fraction patients had a higher risk of biochemical recurrence. Based on the differential genes of plasma cells, plasma cell infiltration status of PCa patients were divided into two independent molecular subtypes(subtype 1 and subtype 2). Subtype 1 tends to be immunosuppressive plasma cells infiltrating to the PCa region, with a higher likelihood of biochemical recurrence, more active immune microenvironment, and stronger immune escape potential, leading to a poor response to immunotherapy. Subsequently, 10 characteristic genes of plasma cell subtype were screened out by three machine learning algorithms. Finally, an artificial neural network was constructed by those 10 genes to predict the plasma cell subtype of new patients. This artificial neural network was validated in an independent validation set, and the similar results were gained.

**Conclusions:**

Plasma cell infiltration subtypes could provide a potent prognostic predictor for prostate cancer and be an option for potential responders to prostate cancer immunotherapy.

## Background

Prostate cancer (PCa) is one of the most common male malignancies worldwide and the second leading cause of cancer-related deaths in men (1). Patients with PCa benefit only modestly from current treatments. Despite radical prostatectomy or androgen deprivation therapy, tumor recurrence and progression are still unavoidable in some patients (2, 3). In recent years, the emerging immunotherapy has shown encouraging effects in a series of cancers (4). The slow growth of PCa and the presence of a variety of tumor-associated antigens as potential targets seem to imply that immunotherapy could become a hope of treatment for PCa (5). However, only a small proportion of PCa patients benefit from immunotherapy, and clinical heterogeneity among these patients remains difficult to explain (6).

Previous studies have shown that infiltrating immune cells into tumors plays a crucial role in tumor occurrence and development, and also has a huge impact on the effectiveness of patient treatment (7). Highly infiltrating immunosuppressive cells such as M2 tumor-associated macrophages (M2-TAMs) and regulatory T cells (Tregs) have been proved to be an important reason for poor prognosis and ineffective treatment in PCa patients (8–10). Some cells previously thought to have anti-tumor effects, such as CD8+T cells, have also been shown to promote lymph node metastasis of PCa (11). Therefore, analysis of the infiltrating immune cells in PCa may help explain the poor prognosis and treatment failure of patients. In recent years, with the deepening of research on the humoral immune system, the role of B cells in the tumor microenvironment has been emphasized. Plasma cells, terminally differentiated B cells, act as antibody “factories” for normal physiological functions and can produce antibodies based on tumor-associated antigens to resist tumors (12). Pan-cancer analysis has shown that plasma cell-associated genes are one of the strongest positive prognostic factors in tumors (13). In the study of malignant tumors such as gastric cancer, metastatic melanoma, and non-small cell lung cancer, increased levels of plasma cell infiltration predict better prognosis and immunotherapy response of patients (14–16). However, studies in cancers such as breast cancer, primary melanoma, and cervical cancer have yielded opposite conclusions (17–19). These contradictory results suggest that plasma cells perform different functions in different malignant tumors. Previous studies have suggested that IgA-secreting plasma cell subtypes in PCa can block anti-tumor T cell responses and exert immunosuppressive effects. Elimination of such cells enhances the efficacy of immune-related therapies (20). The role and mechanism of plasma cells in PCa need to be further elucidated. Therefore, it is urgent to explore the clinical significance and biological function of plasma cells in PCa.

In this study, transcriptome analysis was performed on PCa patients to assess their fractions of plasma cell infiltration. Based on plasma cell characteristic genes, PCa patients were divided into two subtypes with different clinical features, tumor prognosis, and functional annotation. The differences in tumor microenvironment, gene mutations, and immune escape ability between the two subtypes were analyzed to further determine the benefit of immunotherapy between the two subtypes. Finally, an artificial neural network (ANN) to quickly distinguish the two plasma cell subtypes in PCa patients was constructed by a variety of artificial intelligence algorithms and validated in a combined cohort consisting of multiple datasets. Our study aimed to propose a novel plasma cell-based molecular subtyping to provide a new option for individualized survival prediction and treatment options for PCa patients.

## Materials and methods

### Patients data collection

The gene expression profiles of patients were obtained from The Cancer Genome Atlas (TCGA) and the Gene Expression Omnibus(GEO), which served as discovery cohort and validation cohort, respectively. Patients with incomplete follow-up information, normal and metastatic tissue samples were excluded. Finally, the FPKM RNA-seq and clinical information of 481 PCa patients were collected from the TCGA database(https://portal.gdc.cancer.gov/), gene expression data of 449 PCa patients were collected from GSE70768 (21), GSE70769 (21), GSE116918 (22) in the GEO database(https://www.ncbi.nlm.nih.gov/geo/). The platform used by the GEO queue is shown in the **Supplementary Table 1**. The FPKM of the TCGA cohort was converted to TPM according to the previous method of Bo Li et al. (23). The previous approach was used to remove batch effects and merge the GEO cohorts (24, 25). For data standardization, both TCGA and GEO cohorts were processed by log (x+1). In addition, somatic mutation data based on the whole exon sequencing platform was also downloaded from the TCGA database. The demographic information and follow-up data of 930 PCa patients are shown in **Table 1**.

**Table 1:** Demographics and clinicopathological features of PCa patients in the TCGA and GEO cohort.

Characteristics	TCGA ( N = 481 )	GEO ( N = 449 )	Total ( N = 930 )
**Age**			
<=60	216 (23.23%)	83 (8.92%)	299 (32.15%)
>60	265 (28.49%)	276 (29.68%)	541 (58.17%)
NA	0 (0%)	90 (9.68%)	90 (9.68%)
**Gleason score**			
5	0 (0%)	2 (0.22%)	2 (0.22%)
6	45 (4.84%)	77 (8.28%)	122 (13.12%)
7	241 (25.91%)	239 (25.70%)	480 (51.61%)
8	58 (6.24%)	65 (6.99%)	123 (13.23%)
9	133 (14.30%)	64 (6.88%)	197 (21.18%)
10	4 (0.43%)	2 (0.22%)	6 (0.65%)
**BCR state**			
0	415 (44.62%)	329 (35.38%)	744 (80.00%)
1	66 (7.10%)	120 (12.90%)	186 (20.00%)
**BCR time**			
Mean±SD	981.69 ± 767.23	1833.28 ± 972.80	1392.83 ± 970.46
**T stage**			
T0	0 (0%)	1 (0.11%)	1 (0.11%)
T1	0 (0%)	51 (5.48%)	51 (5.48%)
T2	182 (19.57%)	156 (16.77%)	338 (36.34%)
T3	282 (30.32%)	210 (22.58%)	492 (52.90%)
T4	10 (1.08%)	5 (0.54%)	15 (1.61%)
NA	7 (0.75%)	26 (2.80%)	33 (3.55%)
**N stage**			
N0	333 (35.81%)	——	333 (35.81%)
N1	77 (8.28%)	——	77 (8.28%)
NA	71 (7.63%)	449 (48.28%)	520 (55.91%)
**Progression**			
CR+PR	301 (32.37%)	——	301 (32.37%)
PD+SD	62 (6.67%)	——	62 (6.67%)
NA	118 (12.69%)	449 (48.28%)	567 (60.97%)

### Calculation of plasma cell relative fractions based on gene expression

In this study, the CIBERSORT algorithm served to estimate the relative fractions of immune cells in PCa patients. The CIBERSORT algorithm is a linear support vector regression deconvolution algorithm based on machine learning, which is better than the traditional deconvolution method for estimating infiltrating immunity (26). The LM22 reference files were obtained from the original paper, and TCGA and GEO data were converted into a format that is suitable for CIBERSORT algorithm analysis. The relative fractions of plasma cells and 21 other immune cells in PCa samples were obtained by the CIBERSORT algorithm.

### Tumor immune microenvironment patterns of PCa

The ESTIMATE algorithm was used to evaluate stroma and immune cell scores in tumor samples. After evaluating the immune score (immune cell abundance) and stromal score (stromal cell abundance), the ESTIMATE algorithm can further calculate the ESTIMATE score (non-tumor cell abundance) and tumor purity (27). In addition, 29 immune signature gene sets that reflect tumor immune activity were obtained from the Molecular Signatures Database (28) and quantified by single-sample Gene Set Enrichment Analysis (ssGSEA). ConsensusClusterPlus (29) was then used for cluster analysis, K-means clustering algorithm with 1-Spearman correlation distance based on the machine learning algorithm, the empirical cumulative distribution function(CDF) map, and clustering heat map were used to determine the optimal clustering number. Patients with PCa were divided into different cluster centers and named according to immunoactive subtypes.

### Variation analysis between high and low plasma cell fractions groups

According to the median of plasma cell fractions, PCa patients were divided into high plasma cell group and low plasma cell group. Limma was used for differential gene analysis (30). Meet |logFC|>1 and FDR<0.01 genes were differential genes (DEGs). For the enrichment of gene set function analysis, the KEGG rest API (https://www.kegg.jp/kegg/rest/keggapi.html) and org. Hs. Eg. Db (version 3.1.0) were utilized to obtain the results of gene set enrichment by R software package ClusterProfiler(Version 3.14.3). The protein-protein interaction(PPI) network of 25 DEGs was constructed from the STRING database (https://cn.string-db.org/).

### Molecular subtypes of PCa based on DEGs

Similar to the ssGSEA clustering mentioned above, the K-means algorithm based on machine learning was used for the unsupervised clustering of 25 DEGs, and the optimal clustering number was determined by CDF map and clustering heat map. The clinicopathological parameters in various clusters were compared to further explore the association between plasma cell subtypes and clinical characteristics of PCa patients. Furthermore, the clinical characteristics of different clusters were compared to explore the association between plasma cell subtypes and clinical characteristics of PCa patients.

### Gene set variation analysis

The R software package GSVA (31) was used to calculate the enrichment score of each sample in the Gene Set. The c2.cp.kegg.v7.4.symbols.gmt gene set from the Molecular Signatures Database (28) was downloaded to evaluate relevant pathways and molecular mechanisms and got the enrichment score of each sample in each gene set. Limma (30) was further contributed to analyzing the differences in KEGG pathway enrichment score between the two plasma cell subtypes. |log2FC|>0.15 and FDR<0.01 KEGG pathway was considered as a significantly different molecular pathway.

### Immune evasion score and prediction of immunotherapy response

The Tumor Immune Dysfunction and Exclusion (TIDE) Algorithm, developed based on tumor immune evasion mechanism, can predict patient response to immunotherapy through the interaction between tumor gene expression data and T cell infiltration level. Multiple immune characteristics, such as Immune checkpoint, MDSCs, CAFs, and TAMs, were included, and their effectiveness was verified by large-scale immunotherapy data (32, 33). TIDE scores in the python programming environment were used for prediction in this study.

### Machine learning method to select plasma cell subtype pivotal features

The Scikit-Learn (Sklearn), an open-source machine learning library, supports both supervised and unsupervised learning, applying to machine learning feature filtering and ANN construction. It provides a variety of tools for model fitting, data preprocessing, model selection, model evaluation, and many other utilities (34). Firstly, 25 DEGs expression matrices of 481 PCa patients in the TCGA cohort were preprocessed by the StandardScaler function. Plasma cell subtypes were used as labels, the 10-Fold cross-validation method was adopted, and the area under the ROC curve(AUC) was used as assessment criteria. Three representative machine learning models (Random Forest, SVM, XGboost) were used for recursive feature elimination to select plasma cell subtype characteristic genes. The genes were visualized by a Venn diagram after feature selection by the three machine learning models, and the intersection genes were considered to be pivotal features of plasma cell subtype prediction.

### Construction of ANN for plasma cell subtype prediction

Firstly, we meticulously designed an ANN with three fully connected layers except for the input layer and output layer, and the number of neurons in each layer was (64,8,64), respectively. The widely used Relu function was selected as the activation function (35), and the Adam optimizer was selected as the optimizer (36). The initial learning rate was set as 0.01, and the L2 penalty parameter was set as 0.0001. The online tool NN-SVG (http://alexlenail.me/NN-SVG) was adopted to visualize the network structure. The gene expression data were preprocessed by the StandardScaler function. Finally, after the ANN was trained iteratively, the prediction performance of our ANN was evaluated through the ROC curve and the confusion matrix of the verification set. At the same time, accuracy and other model evaluation indexes were calculated according to the confusion matrix. The TCGA queue trained ANN model file(.pickle, **Supplementary File** ANNmodelpred.rar) was saved through the Pickle module, which can be easily available to predict plasma cell subtypes of unknown data.

### Statistical analysis

Log-rank tests were used to estimate the association between survival outcomes in different groups of PCa patients. The Mann-Whitney test and Chi-square test were used to assess differences between the continuous and categorical groups, respectively. The correlation test was based on Pearson analysis, P-value <0.05 and |R|> 0.3 was considered to be significantly correlated. SPSS 25.0 (SPSS Company in Chicago, Illinois, USA) and R4.1.2 software were used for statistical analysis. Double tail P-value<0.05 was considered statistically significant. Report hazard ratios (HRs) and 95% confidence intervals (CI) as necessary.

## Results

### Relationship of plasma cell fractions and clinical features and tumor microenvironment patterns

**Figure 1:**
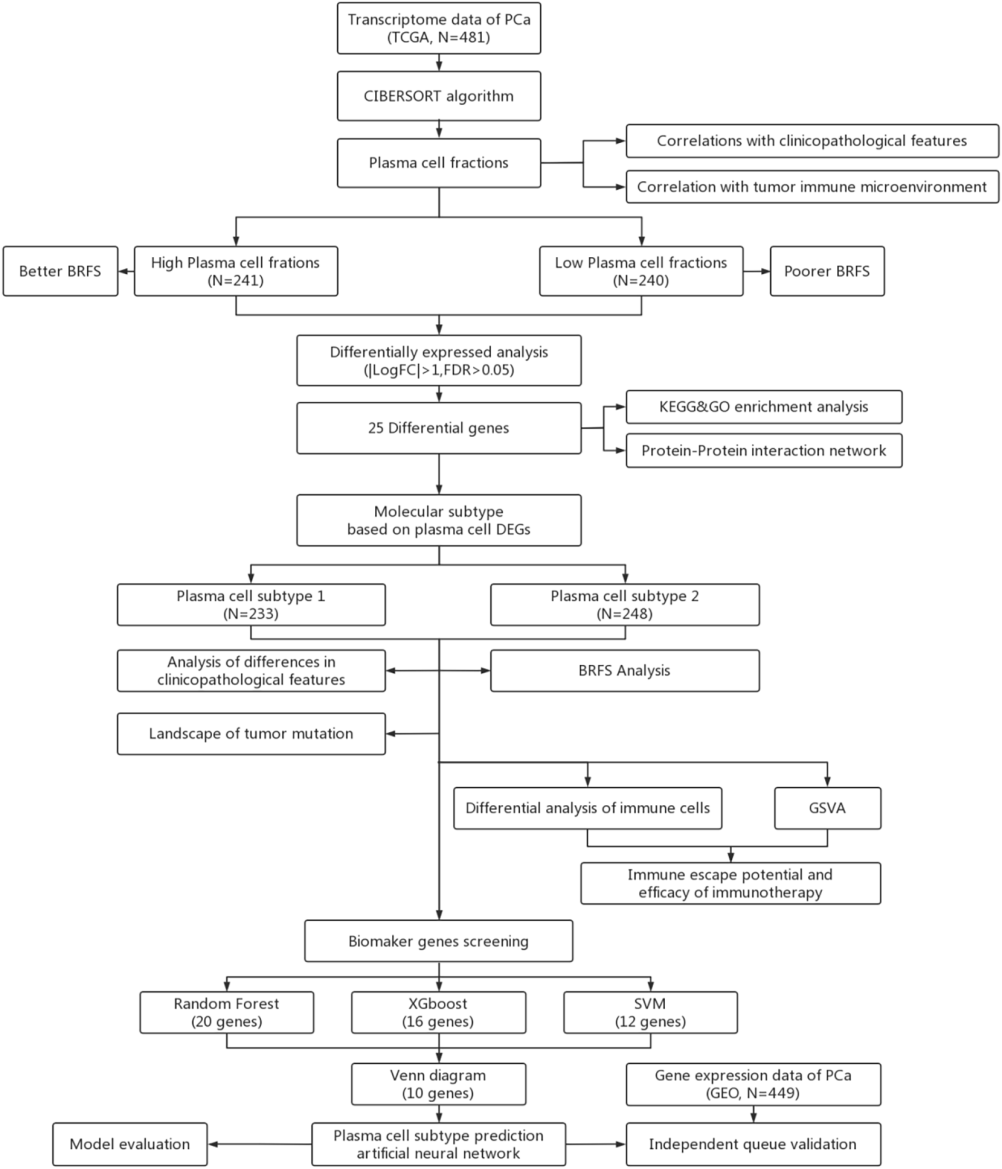
Workflow for analysis of plasma celle subtypes using artificial intelligence algorithms, (BRFS, Biochemical recurrence-free survival. DEGs, Differentially expressed genes; GSVA, Gene set varation analysis).

**Figure 2:**
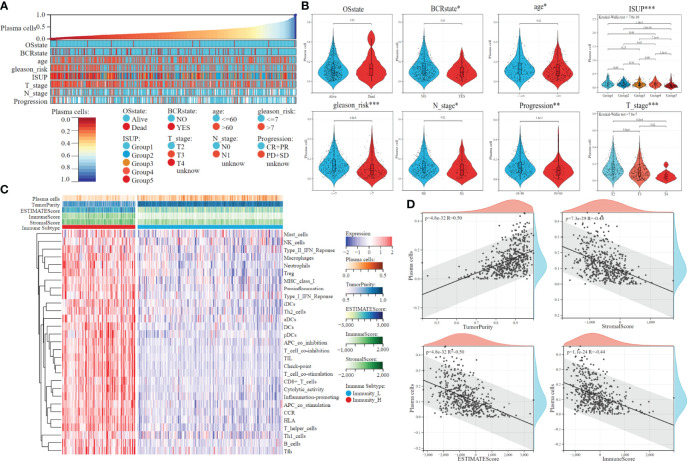
The clinical features and tumor microenvironment patterns associated with the plasma cell fraction in PCa patients. **(A)** The overview of the association between plasma cell fraction and clinical features of patiens. **(B)** Violin plots of plasma cell fraction in individual samples of PCa patiens, stratified by clinical features. **(C)** The immune subtypes of PCa patiens were categorized on the basis of the overall immune activity. **(D)** Correlation analysis between plasma cell fraction and the tumor purity, stromal score, immune score and ESTIMATE score evaluated by ESTIMATE algorithm. *P < 0.05, **P < 0.01, ***P < 0.001.

The overall flow chart of this study is shown in **Figure 1**. First, the content of 22 immune cells in 481 patients with PCa was calculated by the CIBERSORT algorithm, and then plasma cells were inversely ordered to explore the association between plasma cell fractions and clinical features (**Figure 2A**). Plasma cell fractions were significantly increased in biochemical recurrence-free (P<0.05), age<60 (P<0.05), N0 stage (P<0.05), CP+RP (P<0.01), Gleason<=7 (P<0.001), and in both T stage and ISUP stage, plasma cells in patients with high grade were significantly lower than those in patients with low grade (P < 0.001, **Figure 2B**).

The ssGSEA algorithm was used to quantify the overall characteristic level of immune activity in a single patient. Then, 29 immune activity characteristics of 481 patients with PCa were used for unsupervised clustering by the K-means algorithm. The optimal cluster number of CDF distribution map and a consistent heat map was 2 **(Supplementary Figures 1A, B**), that is, 481 patients could be divided into the high immunoactivity group (N=161) and the low immunoactivity group (N=320). The heat map was used to visualize the immune characteristics (**Figure 2C**). Tumor purity, ESTIMATE score, immune score, and stromal score were evaluated by the ESTIMATE algorithm. There was a significant negative correlation between plasma cell fractions and the ESTIMATE score, immune and stromal score, which indicated that the infiltration levels of immune and stromal cells decreased with the increase of plasma cell fractions; there was a significant positive correlation between plasma cell fractions and tumor purity (**Figure 2D**). Then we compared the fractions of plasma cells in the low immunoactivity group and found that the fractions of plasma cells in the low immunoactivity group were significantly higher than that in the high immunoactivity group (**Supplementary Figure 1C**). In addition, we also explored the correlation between plasma cells and other 20 types of immune cells (T cells CD4 naive was 0 in all samples, which was removed below), and found that the content of most immune cells was negatively correlated with plasma cell fractions. T cells CD4 memory resting showed a significant negative correlation with plasma cells (R=-0.48, P=9.3e-30). There was also a negative correlation between Dendritic resting, T cell regulatory, Macrophages M1, Macrophages M2, and plasma cells, respectively (P<0.001, **Supplementary Figure 1D**).

### Plasma cell fraction and DEGs identification

**Figure 3:**
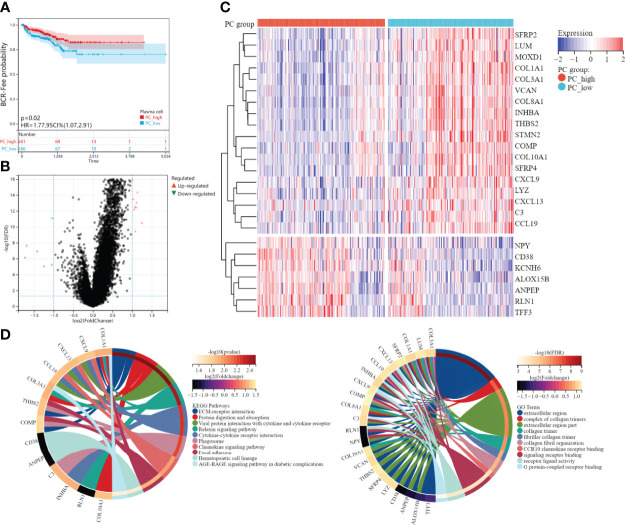
Survival analysis and differential expression between high and low plasma cell fraction groups. **(A)** The higher plasma cell fraction group had better biochemical recurrence-free survival. **(B)** Volcano plots of DEGS between high and low plasma cell fraction groups. **(C)** The DEGs heatmap showed the expression levels between two groups. Red and blue represented high and low expressions, respectively. **(D)** The functional enrichment analyses of DEGs, KEEG enrichment analysis is on the left and GO enrichment analysis is on the right.

According to the median plasma cell fractions, 481 patients were divided into the high plasma cell group and the low plasma cell group. K-M survival analysis showed that the low plasma cell group had significantly lower biochemical recurrence-free time than the high plasma cell group (HR=1.77, p =0.02, **Figure 3A**). Combined with the negative correlation between plasma cells and other immune cells mentioned above, it may indicate that the immune microenvironment in PCa is distinguished from those of these tumors. Therefore, we further explore the molecular subtypes of plasma cells.

Differential expression analysis was conducted between the high plasma cell group and the low plasma cell group, and 25 DEGs were identified, among which 18 genes were significantly up-regulated and 7 genes were significantly down-regulated in the low plasma cell group (**Figures 3B, C**). In addition, we analyzed the correlation between 25 DEGs and 21 kinds of immune cell fractions, and found that 25 DEGs were significantly correlated with plasma cell fraction (**Supplementary Figure 2A**). Then, the KEGG and GO enrichment analysis was performed for the differential genes, and some pathways and biological pathways were visualized (**Figure 3D**). The enriched KEGG pathway is mainly related to cell receptor interactions, such as ECM-receptor interaction, Viral protein interaction with cytokine and cytokine receptor, and Cytokine-cytokine receptor interaction. The enrichment of the GO term is mainly concentrated in the extracellular region, such as the extracellular region and extracellular region part. KEGG and GO enrichment analysis results were correlated with the biological functions of plasma cells. Finally, we constructed a PPI network diagram for 25 DEGs(**Supplementary Figure 2B**).

### Different clinical and molecular characteristics in the two plasma cell molecular subtypes

**Figure 4:**
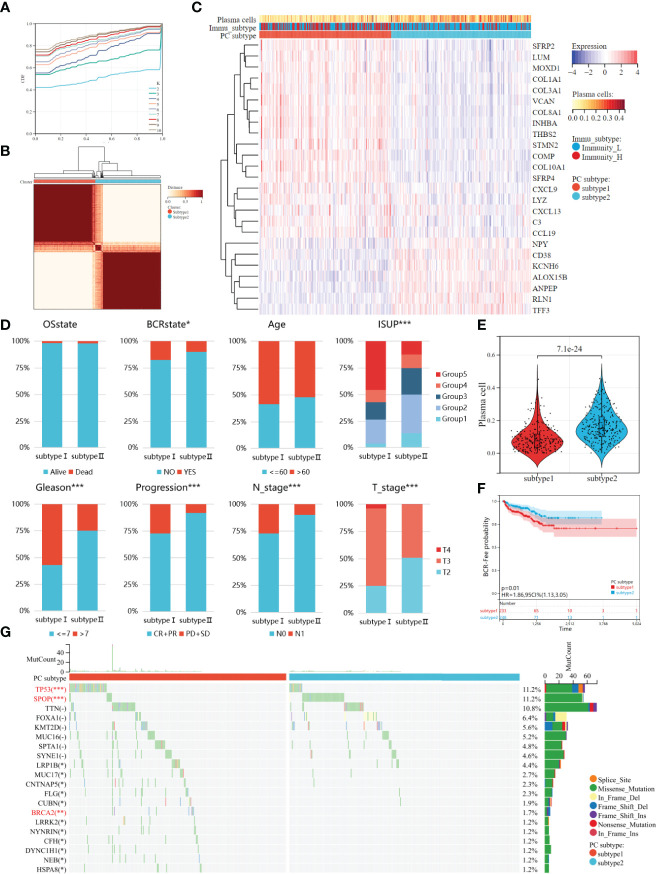
Two molecules subtypes of plasma cell with different clinical characteristics, biochemical recurrence probability, and mutant landscape. **(A)** The CDF curves of the consensus score from k = 2 to 10. **(B)** The Consensus clustering matrix when the best K = 2. **(C)** The heatmap of the expression patterns of 25 DEGs, with red indicating high expressions and blue indicating low expressions. **(D)** Comparison of clinical features between two plasma cell subtypes. **(E)** Kaplan–Meir survival analysis exhibited significantly worse BCR-free survival probability in plasma cell subtype 1. **(F)** Plasma cell fraction was higher in plasma cell subtype 1. **(G)** Waterfall plots showed the top 20 mutated between plasma cell subtype. *P < 0.05, **P < 0.01, ***P < 0.001.

The 25 DEGs were used for unsupervised clustering to identify novel molecular subtypes. According to the CDF curve and consistent heat map, the optimal cluster is determined as 2 (**Figures 4A, B**). Therefore, all PCa patients were divided into two groups, plasma cell subtype 1 (N=233) and plasma cell subtype 2 (N=248), respectively, and the gene expression and immune activity of the two subtypes were visualized by a heat map (**Figure 4C**). We then compared the clinical features of the two plasma cell subtypes, and found that ISUP grading, Gleason score (between <=7 and >7), disease progression, and pathological T and N stage were significantly different (P<0.001), and there was also a significant difference in the number of biochemical recurrences (P<0.05). There was no significant difference between age and survival state (**Figure 4D**). We compared plasma cell fractions between the two subtypes, and plasma cell fractions of subtype 1 were significantly lower than that of subtype 2 (P<0.001, **Figure 4E**). K-M survival analysis showed that plasma cell subtype 1 had worse biochemical recurrence-free survival than plasma cell subtype 2 (HR=1.86, P<0.01, **Figure 4F**). Then, the ESTIMATE algorithm was used to calculate the microenvironments of two plasma cell subtypes. There were higher ESTIMATE scores, immune scores, and stromal scores, however lower tumor purity in plasma cell subtype 1(all P<0.001, **Supplementary Figure 3A**). In addition, we explored the relationship between plasma cell subtypes and immunoactive, and found that there were more high immunoactive groups in plasma cell subtype 1, which may indicate that plasma cell subtype 1 has higher immunoactivity (P<0.001, **Supplementary Figure 3B**). Finally, we visualized the mutation landscape of the top 25 genes with the largest number of mutations in PCa and performed a Chi-square test on the frequency of mutations between the two groups to explore differences in gene mutations among different subtypes (**Figure 4G**). The mutation frequency of TP53, BRCA2, MUC7, CNTNAP5, FLG, etc. in plasma cell subtype 1 was significantly higher than that in plasma cell subtype 2, while the mutation frequency of SPOP was significantly lower than that in plasma cell subtype 2. In previous studies on PCa, TP53 and BRCA2 mutations have been recognized as adverse prognostic factors (37–40). There was no significant correlation between SPOP mutation and prognosis of PCa, but studies have shown that it can improve the sensitivity of PCa to Abiraterone (41, 42).

### Plasma cell molecular subtypes immune escape and response rate of immunotherapy

**Figure 5:**
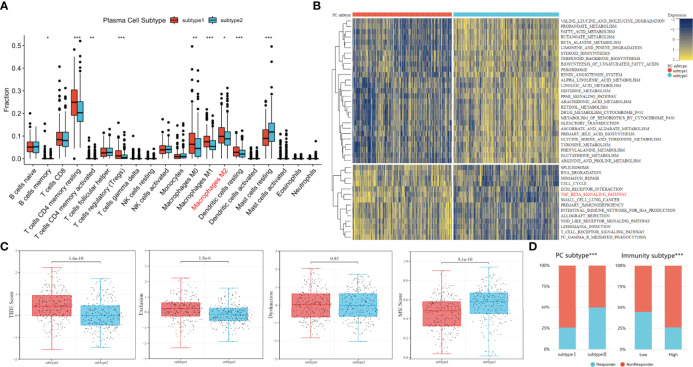
Analysis of immune escape potential and efficacy of immunotherapy between plasma cell subtypes. **(A)** Comparisons of the fraction of 20 immune cells in two plasma cell subtypes. **(B)** Heatmap illustrated the enrichment scores of 41 differentially enriched molecular pathways evaluated by GSVA analysis between plasma cell subtype 1 and 2. Yellow and blue represented high and low enrichment scores, respectively. **(C)** TIDE, Exclusion, Dysfunction and MSI score different plasma cell subtypes. **(D)** Comparisons of the proportions of nonresponders and responder to immunotherapy among different classification methods. (Left: plasma cell subtype, Right: immune subtype). *P < 0.05, **P < 0.01, ***P < 0.001.

Previous studies have shown that immune cells can promote tumor immune escape through cross-talk and change the proportion of immune cells in tumor microenvironment (43, 44). Therefore, the CIBERSORT algorithm was used to compare the content of 20 other immune cells between the two subtypes. The Dendritic cells resting, Macrophages M2, Macrophages M1, Macrophages M0, Tregs, T cells CD4 memory, T cell CD4 memory resting and B cell memory were more abundant in plasma cell subtype 1. Only Mast cells resting (except plasma cells) were highly expressed in plasma cell subtype 2. (all P<0.05, **Figure 5A**). Our previous studies of M2 macrophages have shown that PCa patients with high M2 macrophage infiltration are less sensitive to immunotherapy (10). This may suggest higher immunosuppression and greater immune escape potential in plasma cell subtype 1.

To further explore functional enrichment among plasma cell subtypes, the GSVA was used to further explore the molecular pathways and potential mechanisms between plasma cell subtypes. A total of 41 differentially enriched molecular pathways were identified, among which 27 were significantly down-regulated and 14 were significantly up-regulated in plasma cell subtype 1. The significantly enriched pathways were visualized by a heat map (**Figure 5B**). The down-regulated pathways in plasma cell subtype 1 are mainly related to the metabolism of a variety of substances, such as PROPANOATE_METABOLISM, FATTY_ACID_METABOLISM, BUTANOATE_METABOLISM, etc. The significantly up-regulated pathways in plasma cell subtype 1 are related to cell cycle regulation (CELL_CYCLE), the transmission of genetic information (SPLICEOSOME, RNA_DEGRADATIO) and immune regulation (ECM_RECEPTOR_INTERACTION, TGF_BETA_SIGNALING_PATHWAY, T_CELL_RECEPTOR_SIGNALING_PATHWAY), etc. Previous studies have described a potentially unique immunosuppressive plasma cell subset unique to PCa that responds to the TGFβ pathway and inhibits CD8+T cell-mediated tumor immunity (20, 45). In addition, the TGFβ pathway has also been confirmed by multiple studies that the TGFβ pathway is related to the immune evasion mechanism of tumors, and tumor cells can use the TGFβ pathway to avoid immune monitoring of lymphocytes (46, 47). Therefore, we inferred that plasma cell subtype 1 may be more immunosuppressive plasma cells, and its immune evasion potential may be stronger.

The TIDE algorithm was to evaluate the immune evasion potential of 481 patients with PCa. A higher TIDE score means a higher possibility of immune evasion. The TIDE score of plasma cell subtype 1 was significantly higher than that of plasma cell subtype 2 (P<0.001, **Figure 5C**). In addition, higher TIDE scores were generally associated with poorer prognostic outcomes, which were similar to previous biochemical recurrence-free survival outcomes. We found that plasma cell type 2 patients had a higher microsatellite instability(MSI) score (P<0.001), while plasma cell subtype 1 had a higher T cell exclusion score (P<0.001), but there was no significant difference in T cell dysfunction between the two subtypes(**Figure 5C**). TIDE score also predicted the likelihood of patients responding to immunotherapy. The proportion of immunotherapy responders in plasma cell subtype 1 was significantly lower than that in plasma cell subtype 2 (25.8% vs. 50.00%, P<0.001, **Figure 5D**). For tumor immunoactivity, the proportion of responders in the low tumor immunoactivity group was significantly higher than that in the high tumor immunoactivity group (44.4% vs. 26.1%, P<0.001, **Figure 5D**). These results may indicate that plasma cell subtypes can predict, to a certain extent, whether patients with PCa respond to immunotherapy.

### Characteristic genes for predicting plasma cell molecular subtypes

**Figure 6:**
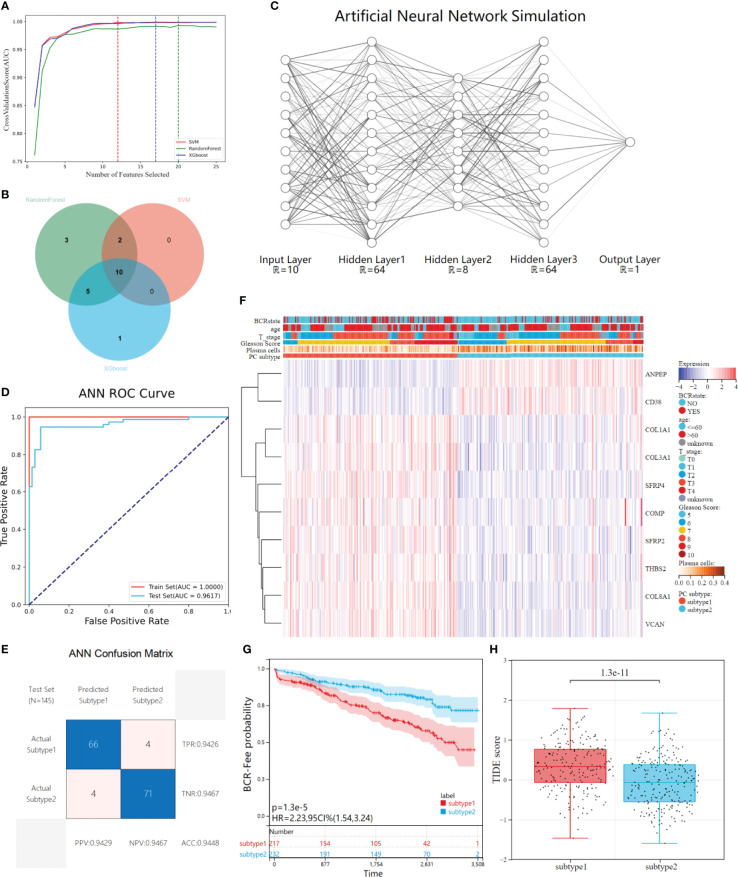
Construction and validation of ANN for plasma cell subtype prediction. **(A)** The average AUC curves of feature screening were obtained by the three machine learning algorithms (Randoms forest, SVM and XGboost) respectively under the 10-fold cross-validation, the dotted line represents the best number of features. **(B)** Venn diagrams shows 10 characteristic genes of plasma cell subtypes shared by the three machine learning models. **(C)** The ANN simulation diagram includes three hidden layers except the input&output layer, and the neurons in each layer are (64,8,64), respectively. Regions larger than 12 neurons are omitted due to plotting limitations. **(D)** The ROC curves of the ANN in distinguishing two subtypes in the train set and test set. **(E)** Confusion matrix and evaluation parameters in test set. **(F)** The GEO independent validatiob queue was predicted using ANN, and the expression of 10 characteristics genes was visualized through heat maps. **(G)** Kaplan-Meier survival analysis showed differences in biochemical recurrence-free probability among plasma cell subtypes in the GEO independently validated cohort. **(H)** There were differences in TIDE scores between the tow plasma cell subtypes in the GEO cohort.

Three representative machine learning algorithms were used to screen DEGs to evaluate key genes for plasma cell subtype prediction. 25 DEGs genes were recursively eliminated by Random Forest, XGboost, and SVM, respectively. Finally, 20, 16, and 12 characteristic genes were identified. The AUC- characteristic curve suggested that the three machine learning models performed superiorly in identifying plasma cell subtypes. After 10 features, the average AUC of 10-fold cross-validation was all greater than 0.95(**Figure 6A**). 10 key genes of plasma cell subtypes were identified by three machine learning algorithms using the Venn diagram (**Figure 6B**).

### Construction and validation of ANN for plasma cell subtype prediction

After feature gene screening, the 481 PCa patients in the TCGA cohort were selected according to their plasma cell subtypes, with stratified random sampling at 7:3 as the training set (N=336) and verification set (N=145), and then used the training set to train the ANN prediction model. The network structure is visualized (**Figure 6C**). Inputting the above 10 key genes of plasma cell subtypes and labeling plasma cell subtypes, the concentration of plasma cell subtypes would be predicted and verified after training. After training, ROC curve analysis showed that our ANN had superior performance in predicting plasma cell subtypes. In the training set, the AUC of plasma cell subtypes was 1.0000, while in the verification set, the AUC was 0.9617(**Figure 6D**). Further, we visualized the confusion matrix of the verification set and evaluated the evaluation indexes of ANN in the verification set. The accuracy of the verification set is 0.9448, and other indexes are shown in the figure(**Figure 6E**).

### Validation of the ANN in an independent cohort

A multi-dataset combined cohort of 449 PATIENTS with PCa was recruited from the GEO database, to validate the clinical application value of plasma cell subtype predictive ANN in different data types and patient populations. The gene expression of 10 plasma cell subtypes from 449 patients was extracted, and then used our trained ANN to predict the subtypes of the patients. 449 patients were divided into 217 plasma cell subtype 1 and 232 plasma cell subtype 2, and gene expression and plasma cell subtype were visualized by a heat map (**Figure 6F**).KM analysis showed that the recurrence-free survival rate of plasma cell subtype 1 was significantly lower than that of plasma cell subtype 2 in the GEO combination cohort (HR= 2.23, P<0.001, **Figure 6G**), was consistent with the TCGA cohort. We also compared the clinical features of the two plasma cell subtypes, which could not be compared in the ISUP stage due to lacking primary/second Gleason. There was no difference in age between the two plasma cell subtypes, while there were more patients with high Gleason score, high T stage, and more biochemical recurrence in plasma cell subtype 1 than in plasma cell subtype 2 (all P<0.001, **Supplementary Figure 4A**). The tumor microenvironment patterns in the GEO combination cohort were evaluated and, similar to TCGA results, significantly reduced ESTIMATE, immune and stromal scores for plasma cell subtype 1 and significantly increased tumor purity scores (all P<0.001, **Supplementary Figure 4B**). The abundance of plasma cell subtype 1 immune and stromal cells was lower than that of tumor cells. Meanwhile, the fractions of plasma cells and 21 other immune cells calculated by CIBERSORT algorithm were compared between the two subtypes(**Supplementary Figure 4C**).

The TIDE scores of two plasma cell subtypes in the GEO combined cohort were compared and found that TIDE scores of plasma cell subtype 1 were significantly higher than those of plasma cell subtype 2 (P<0.001, **Figure 6H**). In addition, plasma cell subtype 1 had a higher exclusion score (P<0.001), the MSI score was lower (P<0.001), but there was no significant difference in dysfunction score between the two subtypes (**Supplementary Figure 4D**). Plasma cell subtype 1 is also insensitive to immunotherapy (P<0.001, **Supplementary Figure 4E**), which was consistent with TCGA cohort results. The risk values for biochemical recurrence of 10 characteristic genes in TCGA and GEO cohorts and the AUC values used to distinguish the two plasma cell subtypes were calculated as **Table 2** and **Supplementary Table 2**. Overall, plasma cell subtype 1 was associated with a worse prognosis, a greater immune evasion potential, and a poorer response to immunotherapy in the GEO combined cohort. All of these findings suggest that the novel plasma cell molecular classification we constructed by ANN is robust and can be applied to different patient populations in different cohorts.

**Table 2:** The 10 gense selected by multiple machine learning algorithms (TCGA cohort).

Variables	PC subtype predition AUC (95%CI)	HR (95%CI)	P-value
ANPEP	0.85 (0.82-0.89)	0.89 (0.81-0.97)	5.77E-03
CD38	0.76 (0.72-0.81)	0.80 (0.70-0.91)	4.14E-04
COL1A1	0.80 (0.76-0.84)	1.61 (1.32-1.96)	2.58E-06
COL3A1	0.78 (0.74-0.82)	1.41 (1.15-1.73)	1.19E-03
COL8A1	0.83 (0.79-0.86)	1.42 (1.15-1.75)	8.65E-04
COMP	0.84 (0.80-0.88)	1.35 (1.18-1.56)	1.03E-05
SFRP2	0.80 (0.76-0.84)	1.36 (1.12-1.66)	2.26E-03
SFRP4	0.84 (0.80-0.87)	1.31 (1.12-1.55)	1.01E-03
THBS2	0.84 (0.80-0.87)	1.46 (1.23-1.75)	1.68E-05
VCAN	0.80 (0.76-0.84)	1.36 (1.13-1.65)	1.31E-03

## Discussion

Compared to other types of malignancies, PCa grows slowly, which makes it an ideal candidate for effective immunotherapy (5, 48). Therefore, immunotherapy for PCa has received extensive attention, including checkpoint inhibitors, cytokines, and therapeutic cancer vaccines (49). Currently, anti-PD1/PDL1 and anti-CTLA4 monoclonal antibodies have been tested in the treatment of patients with mCRPC, but the trial results have been disappointing. Only a limited number of patients benefited from immunotherapy, and it is challenging to explain this heterogeneity. In previous clinical studies, patients with PCa were not selectively recruited by molecular omics, or by PD1/PDL1 or CTLA4 expression alone (50–52). Due to the instability of immune checkpoint expression in PCa, it is difficult to be a reliable biomarker for immune checkpoint therapy (10, 53). Therefore, such unselected treatment may lead to the failure of these immunotherapy clinical trials. How to screen out patients with PCa who are more responsive to immunotherapy has become an issue that needs to be focused on in the future.

Based on the above problems, this study provides insight into the relationship between plasma cells and immunotherapy in PCa, and introduced two subtypes according to the characteristic genes of plasma cells. The results showed that although having a higher immune activity, plasma cell subtype 1 has a higher biochemical recurrence probability, higher immune escape potential, and a poorer response to immunotherapy In fact, in studies of other tumors, immune cell activity in the tumor microenvironment has been found to be mostly a positive prognostic factor, and immunoactive patients may be more responsive to immunotherapy (54–56). We found that even with higher immune activity in plasma cell subtype 1, its immunotherapy efficacy was still poor. These results suggest that immune escape may be promoted by a unique mechanism in PCa plasma cells, thereby affecting the efficacy of immunotherapy.

Whether plasma cells can directly mediate immune escape in PCa has not been fully investigated, there is still some evidence that plasma cells interact with the immune microenvironment to induce tumor immune escape. On the one hand, plasma cells can inhibit the activity of effector T cells by releasing immunosuppressive cytokines such as IL-10 and IL-35, thereby weakening the tumor-killing effect of effector T cells (12). On the other hand, plasma cells promote the production of regulatory T cells (Tregs) by secreting TGF-β (12),and promote the production of M2 macrophages (M2-TAMs) by secreting GABA (57)These immunosuppressive cells limit the infiltration of effector T cells into the tumor area (58). Thus, even if the overall immune activity of the tumor is strong, plasma cell-induced T cell anergy and exclusion prevent the immune system from mounting an effective anti-tumor response, inducing the immune escape of the tumor. In addition, plasma cells can interact with Treg cells to transform into immunosuppressive plasma cells (12). Previous study in PCa have shown that the removal of immunosuppressive plasma cells from TRAMP mice receiving immunogenic chemotherapy is able to increase T-cell infiltration in the tumor area, thereby improving the efficacy of immunogenic chemotherapy (20). All the above studies suggest that there is a correlation between plasma cells and immune escape. Immunotherapy targeting plasma cells or combining immunotherapy with anti-plasma cell therapy may be the focus of future PCa research.

This study does, however, have some limitations. First of all, this study used public database analysis of TCGA and GEO cohort, lacking direct evidence to investigate the relationship between plasma cell infiltration and the immunotherapy response rate of PCa. So these findings need to be confirmed by further experiments, which is what we are going to do next. Secondly, ANN is often called the “black box” due to its internal interpretability (59, 60). Currently, it is challenging to determine how to weigh the internal features of a model. In addition, more samples are also required to verify the ANN model. Finally, the number of PCa patients currently receiving immunotherapy is very limited, so the relationship between the plasma cell molecular subtypes we introduced and immunotherapy responsiveness still needs to be validated in future immunotherapy cohorts.

In conclusion, immunotherapy for PCa holds great promise, and screening patients who may benefit from immunotherapy is one of the important tasks. This study divided PCa patients into two different subtypes based on the molecular characteristics of plasma cells, and predicted the response of patients with different subtypes to immunotherapy, providing a potential method for screening immunotherapy-sensitive patients in the future. An ANN for the prediction of plasma cell subtypes was constructed in this study, which was convenient for the clinical application of plasma cell subtypes.

## Conclusions

Plasma cell infiltration subtypes could provide a potent prognostic predictor for prostate cancer and be an option for potential responders to prostate cancer immunotherapy.

## Data availability statement

The datasets presented in this study can be found in online repositories. The names of the repository/repositories and accession number(s) can be found below: https://portal.gdc.cancer.gov/, TCGA-PRAD;https://www.ncbi.nlm.nih.gov/geo/, GSE70768; https://www.ncbi.nlm.nih.gov/geo/, GSE70769;https://www.ncbi.nlm.nih.gov/geo/, GSE116918.

## Author contributions

M-KC, S-CZ was responsible for managing the project, supervising the research process and revising the manuscript. XX, C-XD, and M-RL analyzed data from prostate cancer patients and were major contributors to writing the manuscript. KZ, YL and J-WZ provided methodology ideas and modified the paper in detail. Z-PH, K-YX, H-YL, A-RO, S-XM assisted to complete the data visualization and modified the image details. J-KY, Q-ZZ, W-BG and C-DL discussed and guided the idea of the article. All authors contributed to the article and approved the submitted version.
