# Supplementary material for: Plasma cell subtypes analyzed using artificial intelligence algorithm for predicting biochemical recurrence, immune escape potential, and immunotherapy response of prostate cancer
Source: Front Immunol. 2022 Dec 8;13:946209. doi: 10.3389/fimmu.2022.946209 (PMC9772552; doi:10.3389/fimmu.2022.946209)
Supplement: Supplementary file 1 [file DataSheet_1.pdf]

Supplementary table 1.The GEO queue and the platform used.

| Dataset | Platforms |
|---|---|
| GSE70768 | GPL10558 |
| GSE70769 | GPL10558 |
| GSE116918 | GPL25318 |

Supplementary table 2. The 10 genes from the ANN model(GEO cohort).

| Variables | PC subtype predition AUC (95%CI) | HR (95%CI) | P-value |
|---|---|---|---|
| ANPEP | 0.90 (0.87-0.93) | 0.63 (0.53-0.76) | 6.47E-07 |
| CD38 | 0.78 (0.73-0.82) | 0.67 (0.56-0.81) | 1.96E-05 |
| COL1A1 | 0.72 (0.68-0.77) | 2.10 (1.60-2.74) | 5.24E-08 |
| COL3A1 | 0.77 (0.73-0.82) | 1.73 (1.31-2.28) | 9.47E-05 |
| COL8A1 | 0.84 (0.81-0.88) | 1.75 (1.33-2.31) | 5.32E-05 |
| COMP | 0.73 (0.69-0.78) | 1.43 (1.26-1.63) | 2.60E-07 |
| SFRP2 | 0.78 (0.73-0.82) | 1.54 (1.21-1.95) | 4.17E-04 |
| SFRP4 | 0.83 (0.79-0.87) | 1.39 (1.16-1.66) | 2.75E-04 |
| THBS2 | 0.75 (0.71-0.80) | 1.95 (1.48-2.56) | 2.09E-06 |
| VCAN | 0.84 (0.81-0.88) | 1.61 (1.31-1.98) | 4.84E-06 |
