# Supplementary material for: Plasma cell subtypes analyzed using artificial intelligence algorithm for predicting biochemical recurrence, immune escape potential, and immunotherapy response of prostate cancer
Source: Front Immunol. 2022 Dec 8;13:946209. doi: 10.3389/fimmu.2022.946209 (PMC9772552; doi:10.3389/fimmu.2022.946209)
Supplement: Supplementary file 2 [file DataSheet_2.pdf]

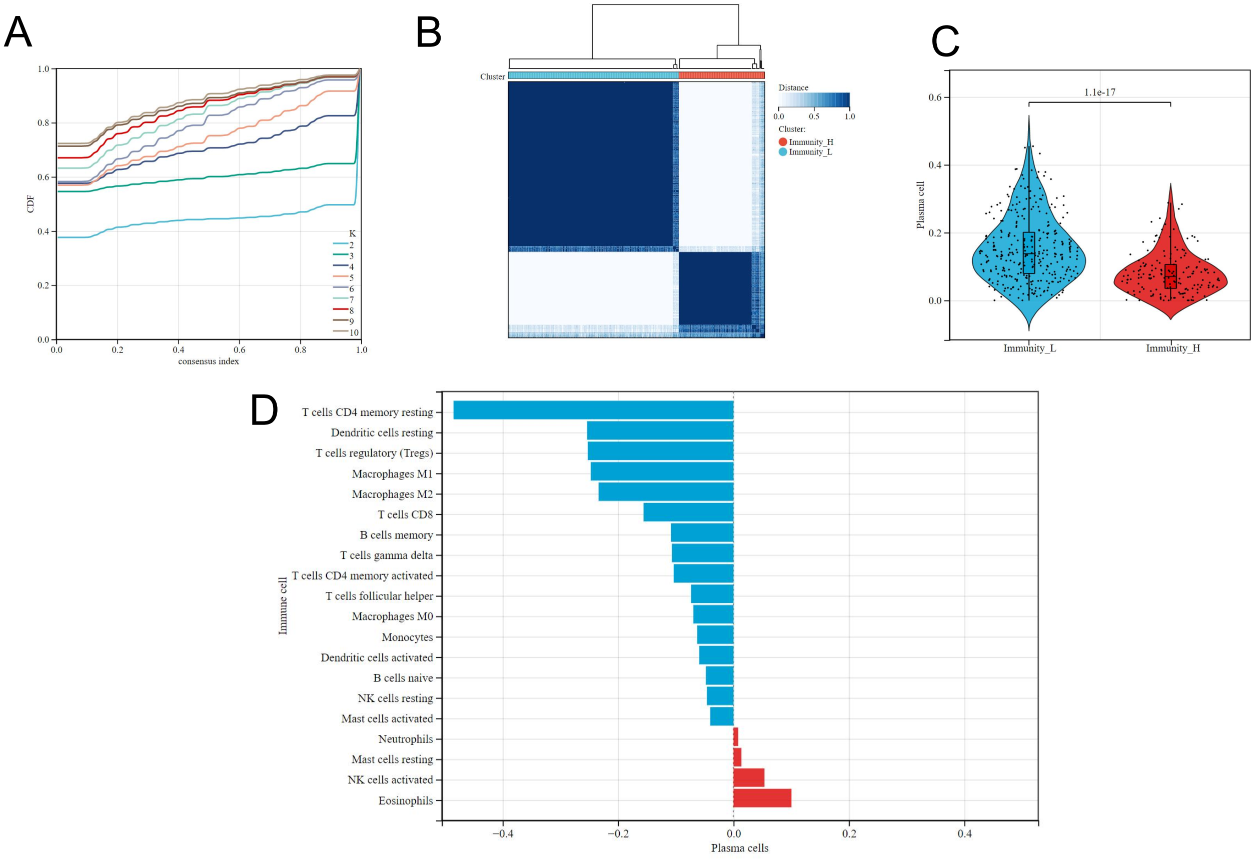

Supplementary Figure 1.
(A)The CDF curves of the consensus score from k = 2 t o 10.
(B)The Consensus clustering matrix when the best k = 2.
(C)There were differences in plasma cell fraction between high and low immunoactivity subtype.
(D)Correlation between plasma cell fraction and other 20 immune cell fractions.

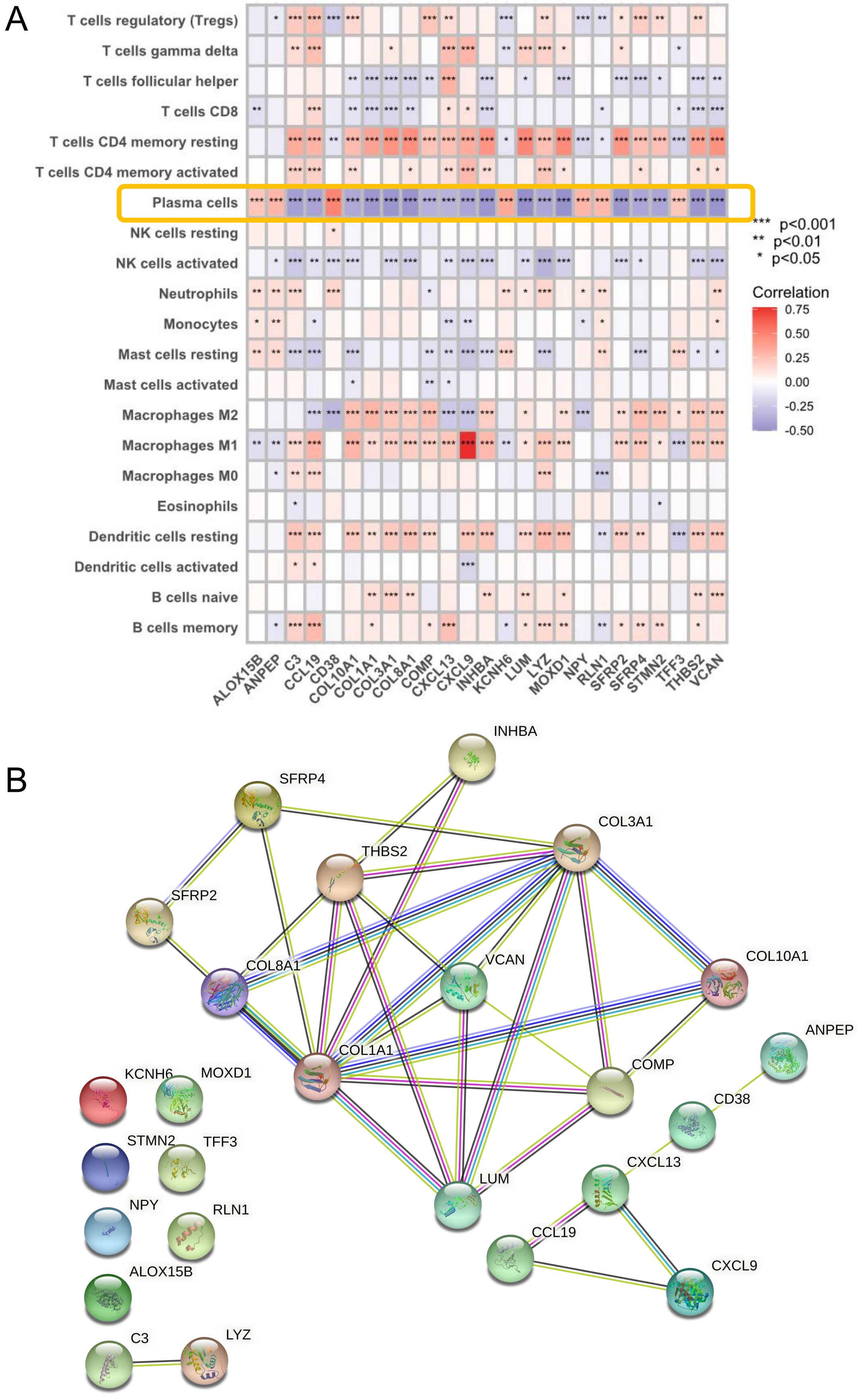

Supplementary Figure 2.
(A)Correlation analysis between DEGs and immune cells fractions. Red meant positive correlation, and blue meant negative correlation.
(B)Protein interaction network of 25 DEGs.

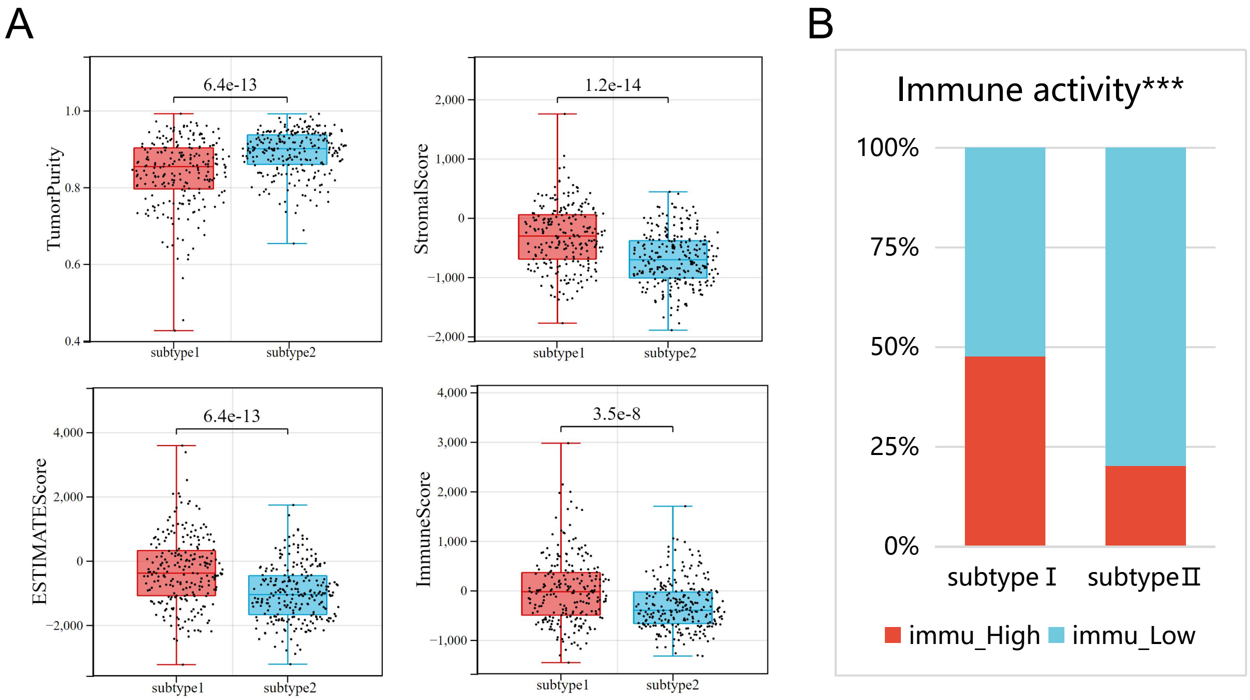

Supplementary Figure 3.
(A)Comparisons of tumor purity, stromal score, immune score and ESTIMATE score between two plasma cell subtypes.
(B)The different proportions of high and low Immune activity tumors in two plasma cell subtypes.

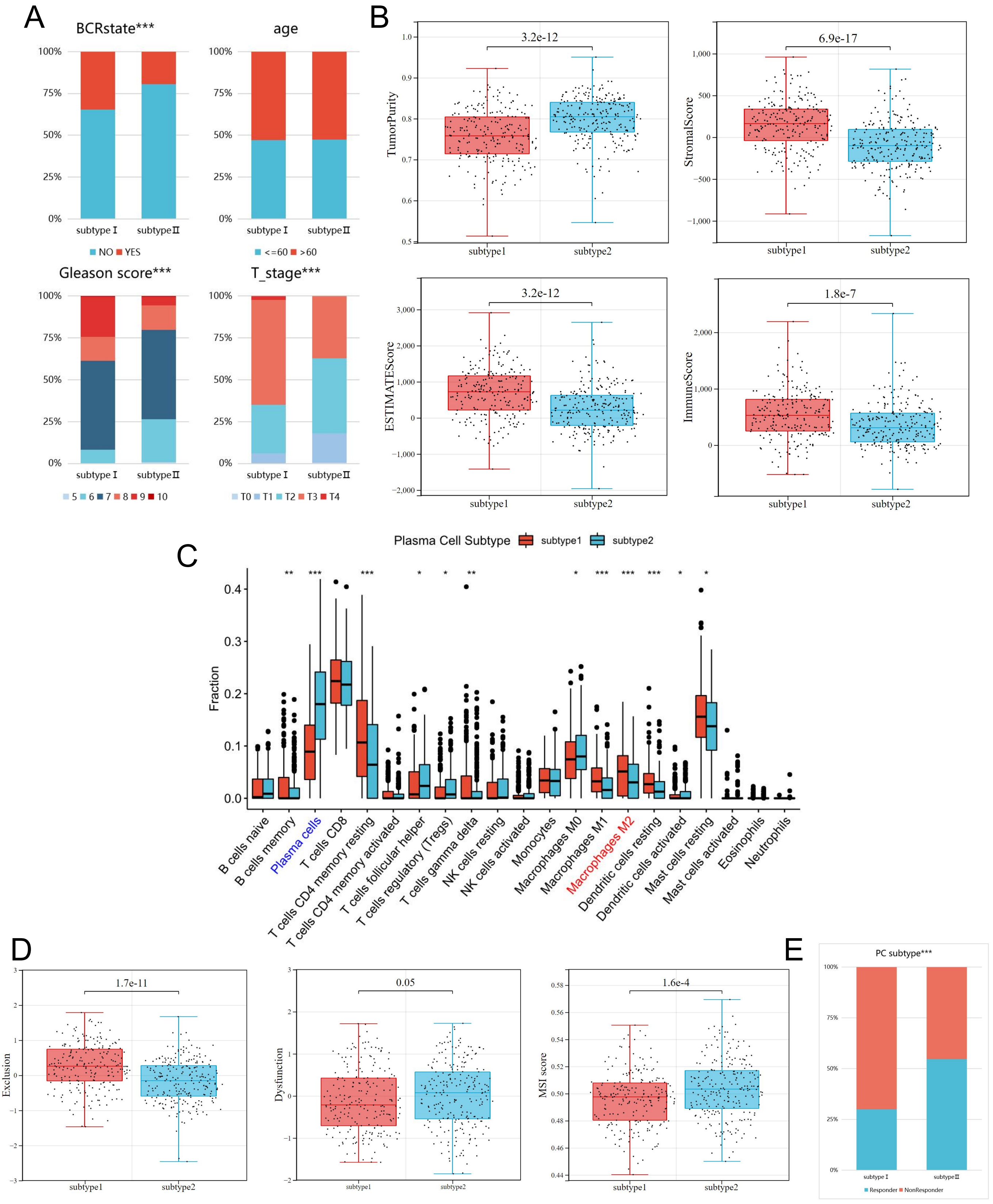

Supplementary Figure 4.

(A)Comparison of clinical features between two plasma cell subtypes.

(B)Comparisons of tumor purity, stromal score, immune score and ESTIMATE score between two plasma cell subtypes.

(C)Comparison of 21 immune cell fractions between plasma cell subtypes.

(D)Exclusion, Dysfunction and MSI score in different plasma cell subtypes.

(E)Comparison of proportion of nonresponders and responders to immunotherapy between plasma cell subtypes
